# Patterns and predictors of recurrence after laparoscopic resection of rectal cancer

**DOI:** 10.3389/fonc.2022.1034838

**Published:** 2022-10-27

**Authors:** Hong Yang, Lei Chen, Xiuxiu Wu, Chenghai Zhang, Zhendan Yao, Jiadi Xing, Ming Cui, Beihai Jiang, Xiangqian Su

**Affiliations:** Key laboratory of Carcinogenesis and Translational Research (Ministry of Education), Department of Gastrointestinal Surgery IV, Peking University Cancer Hospital & Institute, Beijing, China

**Keywords:** rectal cancer, laparoscopic surgery, locoregional recurrence, distant metastasis, salvage surgery

## Abstract

**Purpose:**

This study was designed to evaluate the patterns and predictors of recurrence in patients who underwent laparoscopic resection of rectal cancer.

**Methods:**

Patients with rectal cancer receiving laparoscopic resection between April 2009 and March 2016 were retrospectively analyzed. The association of recurrence with clinicopathological characteristics was evaluated using multivariate analyses.

**Results:**

A total of 405 consecutive patients were included in our study. Within a median follow-up time of 62 months, 77 patients (19.0%) experienced disease recurrence: 10 (2.5%) had locoregional recurrence (LR), 61 (15.1%) had distant metastasis (DM), and 6 (1.5%) developed LR and DM synchronously. The lung was the most common site of metastasis. Multivariate analyses indicated that involved circumferential resection margin (CRM) was the only independent predictor for LR (OR=13.708, 95% CI 3.478-54.026, P<0.001), whereas elevated baseline level of CA19-9 (OR=3.299, 95% CI 1.461-7.449, P=0.032), advanced pN stage (OR=2.292, 95% CI 1.177-4.462, P=0.015) and harvested lymph nodes less than 12 (OR=2.418, 95% CI 1.245-4.695, P=0.009) were independently associated with DM. Patients receiving salvage surgery showed superior 3-year survival compared with palliative treatment after relapse (90.9% vs. 20.5%; P=0.017). The estimated 5-year DFS and CSS for the entire cohort was 80.2% and 83.1%, respectively.

**Conclusions:**

DM was more common than LR after laparoscopic resection of rectal cancer, and there were several clinicopathological factors related to LR and DM. Involved CRM and suboptimal lymph node yield were adverse surgery-related factors of tumor recurrence, which should be paid more attention to during the operation.

## Introduction

Rectal cancer is a common malignant disease worldwide. Tremendous changes have taken place in the treatment strategies of rectal cancer over the past few decades, and the most important innovations were the introduction of total mesorectal excision (TME) and neoadjuvant chemoradiotherapy, which have played a crucial role in the significant improvement of tumor prognosis (1–4). Nevertheless, many patients continue to experience tumor recurrence after curative surgery, ranging from 20.8% to 24.3% (5–7). Therefore, early detection of relapse is critical for successful treatment of recurrent disease. The treatment for recurrent rectal cancer is a clinical challenge, there has been extensive discussion about the efficacy of salvage surgery for the past few years. It has been confirmed that salvage surgery with curative intent can significantly improve survival in patients with recurrence (6–8).

With the development of minimally invasive technology, laparoscopic resection of rectal cancer has been widely performed in recent years. Several studies, including some randomized clinical trials, have verified that laparoscopic surgery for rectal cancer was safe and feasible, with satisfactory short- and long-term outcomes compared with open surgery (9–11). Laparoscopic surgery can provide better surgical vision during resection of rectal cancer, theoretically helping surgeons identify and access the correct anatomic plane more easily. However, laparoscopic surgery has not been proven to reduce the risk of involved circumferential resection margin (CRM) and locoregional recurrence (LR) (9, 10). So far, the patterns and predictors of rectal cancer recurrence, and time to relapse after laparoscopic surgery have not been extensively investigated. Moreover, salvage surgery for recurrent disease following laparoscopic resection of rectal cancer was seldom discussed. The purpose of the present study was to characterize the patterns and predictors of recurrence in patients who underwent laparoscopic resection of rectal cancer.

## Methods

### Patients

All patients with pathologically confirmed rectal adenocarcinoma undergoing laparoscopic curative surgery at the Department of Gastrointestinal Surgery IV, Peking University Cancer Hospital from April 2009 to March 2016 were obtained from a prospectively maintained cancer database. Patients with any of the following conditions were excluded from the present study: (a) patients with simultaneous distant metastases, (b) patients with synchronous tumors or history of other tumors within 5 years, (c) patients undergoing palliative resection, (d) patients undergoing emergent surgery, (e) patients with combined evisceration and (f) patients with incomplete clinicopathological data. This study was approved by the Research Ethics Committee of Peking University Cancer Hospital & Institute.

### Treatment

The treatment strategies were selected according to the location and stage of the disease. Preoperative work-up included digital rectal examination, routine blood testing, serum CEA and CA19-9 levels, and colonoscopy biopsy. The clinical staging was evaluated by means of chest radiography or CT, abdominopelvic CT, and pelvic MRI. Endorectal ultrasound was offered to patients with middle or low rectal cancer. Middle and low rectal cancer was defined as the lower edge of the tumor located less than 10 cm from the anal verge, while high rectal cancer as the lower edge of the tumor between 10 and 15 cm from the anal verge, which was measured by colonoscopy at initial diagnosis.

Patients with locally advanced middle or low rectal cancer (clinical staging T3/T4 or N+) were routinely suggested to have neoadjuvant therapy (NAT), which mainly consisted of long-term radiotherapy and concomitant administration of capecitabine (825 mg/m2 twice daily). The gross tumor volume (GTV) was defined as the primary tumor along with the mesorectum. The clinical target volume (CTV) was considered as GTV plus internal iliac and obturator lymphatic drainage regions, presacral region and pelvic wall. A total dose of 50.6 Gy/22 F (2.3 Gy/F) was delivered to GTV, and a total dose of 41.8 Gy/22 F (1.9 Gy/F) was delivered to CTV. About 6 to 8 weeks after finishing radiotherapy, laparoscopic-assisted surgery was implemented based on TME principle, whereas patients without NAT received TME directly. Patients with stage III and stage II disease with risk factors confirmed by postoperative pathology (poor differentiation, serosal or peritoneal involvement, retrieved lymph nodes less than 12, lymphovascular or perineural invasion, or involved CRM) were suggested to have adjuvant chemotherapy, which was routinely recommended for patients receiving NAT regardless of stage. The most common regimen was 5-fluorouracil-based chemotherapy, including the CAPEOX (intravenous oxaliplatin 130 mg/m^2^ on day 1 plus oral capecitabine 1000 mg/m^2^ twice daily on days 1-14 every 3 weeks), mFOLFOX6 (intravenous oxaliplatin 85 mg/m^2^ plus leucovorin 400mg/m^2^, 5-fluorouracil 2400mg/m^2^ on day 1 every 2 weeks) or capecitabine (1000 mg/m^2^ twice daily on days 1-14 every 3 weeks) alone.

All the operations were implemented by a same group of surgeons using five trocars. Sphincter-preserving or non-preserving surgery was primarily depended on the location of tumor and the surgeon’s judgment in the operation. In this cohort, sphincter-preserving surgery was defined primarily as low anterior resection (LAR). Abdominoperineal resection (APR), extralevator abdominoperineal excision (ELAPE), and Hartmann’s procedure were regarded as non-preserving surgery. For middle or low rectal cancer, TME was performed. For high rectal cancer, the mesorectum and the rectum were divided 5 cm from the tumor lower edge, and a partial mesorectal excision was performed. The rectum was divided by the use of 60 mm endoscopic linear staplers. When performing LAR, the specimen was taken out through a small incision by extending the trocar in the left lower quadrant. Colorectal anastomosis was then performed intracorporeally using a circular stapling device, normally with a diameter of 29 mm. Preventive stoma was implemented selectively in the light of tumor location and exact operative process. When preservation of the anus was infeasible or the levator muscle was invaded, APR or ELAPE was performed. The procedures of perineal operation and colostomy were similar to those applied in previous study (12). Hartmann’s procedure was recommended for elderly and infirm patients with low rectal cancer.

The American Joint Committee on Cancer (AJCC) TNM staging system (eighth edition) was used for pathologic evaluation (13). Two pathologists reviewed the pathologic results independently. Positive CRM was defined as tumor invasion within 1 mm from the incisal edge.

### Follow-Up

Patients were followed up at 3-month intervals in the first 2 years after surgery, at 6-month intervals in the following 3 years, and once a year thereafter. Follow-up assessment included physical examination, routine blood testing and evaluation of CEA and CA19-9 levels. Chest radiography or CT, abdominopelvic CT were conducted every 6 months, and a colonoscopy was carried out annually. LR was defined as histologically or radiologically confirmed tumor relapse within the pelvis, whereas distant metastasis (DM) was defined as tumor relapse in different anatomical sites. Disease-free survival (DFS) was defined as the time from the day of surgery to any type of relapse. Cancer-specific survival (CSS) was measured from the day of surgery to death from the same cancer.

For patients diagnosed with recurrence during follow-up, detailed examinations were conducted to fully evaluate the condition, and multidisciplinary consultation would be introduced to determine whether the patients were eligible for salvage surgery and whether NAT was required. For patients who had lost the opportunity of salvage surgery, chemotherapy, radiotherapy or other palliative treatment were selected.

### Statistical analysis

Continuous data was described as median and range, while categorical data was reported as frequency. Univariate logistic regression was performed to explore the factors associated with LR and DM. DFS and CSS were estimated by Kaplan-Meier survival curves and univariately tested with log rank tests. All variables with potential significance in the univariate analyses were included in the multivariate analyses (based on a P-value <0.1). Multivariate analyses were performed with logistic regression analyses for LR and DM, and with Cox regression analyses for DFS and CSS. SPSS 22.0 software (IBM Corporation, Chicago) was used for statistical analyses. A P-value <0.05 was considered statistically significant.

## Results

### Clinicopathology and treatment of patents

A total of 405 consecutive patients who underwent laparoscopic rectal excision were enrolled in our study. The median age was 60 years (range, 23-85), and 223 (55.1%) were male. Nearly two-third of the patients had mid/low rectal cancer, and 287 (70.8%) underwent sphincter-preserving surgery. Seventeen patients (4.2%) were converted to open surgery by reason of intraoperative adhesion, bleeding or other difficulties during operation. Postoperative complications were observed in 51 patients (12.6%), of whom 10 patients suffered reoperation. The most common complications were pelvic abscess (2.7%) and ileus (3.0%), and the incidence of anastomotic leakage was 1.5%. One patient died suddenly of unknown causes within 30 days after discharge from the hospital. The median hospital stay after surgery was 8 days (range, 3-33). Pathologic evaluation indicated that the proportions of patients with stage-0, I, II and III diseases were 4.0%, 25.6%, 28.4% and 42.0%, respectively. R0 resection was achieved in 393 patients (97.0%), apart from 12 patients with involved CRM. The median number of harvested lymph nodes was 14 (range, 2-43), and 71.1% of resections had at least 12 lymph nodes evaluated. Overall, 115 patients (28.4%) had NAT before surgery, almost all of whom completed the treatment, and 220 (54.3%) received adjuvant chemotherapy after surgery, with the vast majority completing between 4 to 8 cycles. Detailed clinical and treatment characteristics are presented in **Table 1**, and pathological characteristics are summarized in **Table 2**.

**Table 1:** Demographic and treatment characteristics.

	n (%)
Age (years), median	60 (23–85)
Sex	
Male	223 (55.1)
Female	182 (44.9)
ASA	
l	180 (44.4)
ll-III	225 (55.6)
BMI (kg/m2), median	24 (15–33)
Location	
Mid/low	266 (65.7)
High	139 (34.3)
Initial CEA (ng/ml)	
≤5	282 (69.6)
>5	123 (30.4)
Initial CA19-9 (U/ml)	
≤37	371 (91.6)
>37	34 (8.4)
Surgical procedure	
LAR	287 (70.8)
APR	57 (14.1)
ELAPE	57 (14.1)
Hartmann	4 (1.0)
Operation time (min), median	208 (77–468)
Blood loss (ml), median	50 (5–2000)
Conversions	17 (4.2)
Postoperative complications	51 (12.6)
Pelvic abscess	11 (2.7)
Ileus	12 (3.0)
Anastomotic leakage	6 (1.5)
Anastomotic hemorrhage	3 (0.7)
Wound infection	4 (1.0)
Uroschesis	4 (1.0)
Urethral injury	1 (0.2)
Cardiopulmonary and cerebrovascular	10 (2.5)
Reoperation	10 (2.5)
Postoperative LOS (d), median	8 (3–33)
30-day mortality	1 (2.5)
Neoadjuvant therapy	115 (28.4)
Adjuvant chemotherapy	220 (54.3)

ASA, American Standards Association; BMI, body mass index;

CEA, carcinoembryonic antigen; LAR, low anterior resection;

APR, abdominoperineal resection; ELAPE, extralevator abdominoperineal excision;

LOS, length of stay.

**Table 2:** Pathological characteristics.

	n (%)
Tumor differentiation	
Well/moderate	339 (83.7)
Poor	66 (16.3)
pT stage	
T0	16 (4.0)
T1	26 (6.4)
T2	106 (26.2)
T3	209 (51.6)
T4	48 (11.8)
pN stage	
N0	235 (58.0)
N1	95 (23.5)
N2	75 (18.5)
pTNM stage	
0	16 (4.0)
l	104 (25.6)
ll	115 (28.4)
III	170 (42.0)
Harvested lymph nodes, median	14 (2–43)
Lymphovascular invasion	54 (13.3)
Perineural invasion	25 (6.2)
DRM (cm)	
≥1	385 (95.1)
<1	20 (4.9)
CRM (mm)	
>1	393 (97.0)
≤1	12 (3.0)

DRM, distal resection margin; CRM, circumferential resection margin.

### Patterns and predictors of recurrence

**Figure 1:**
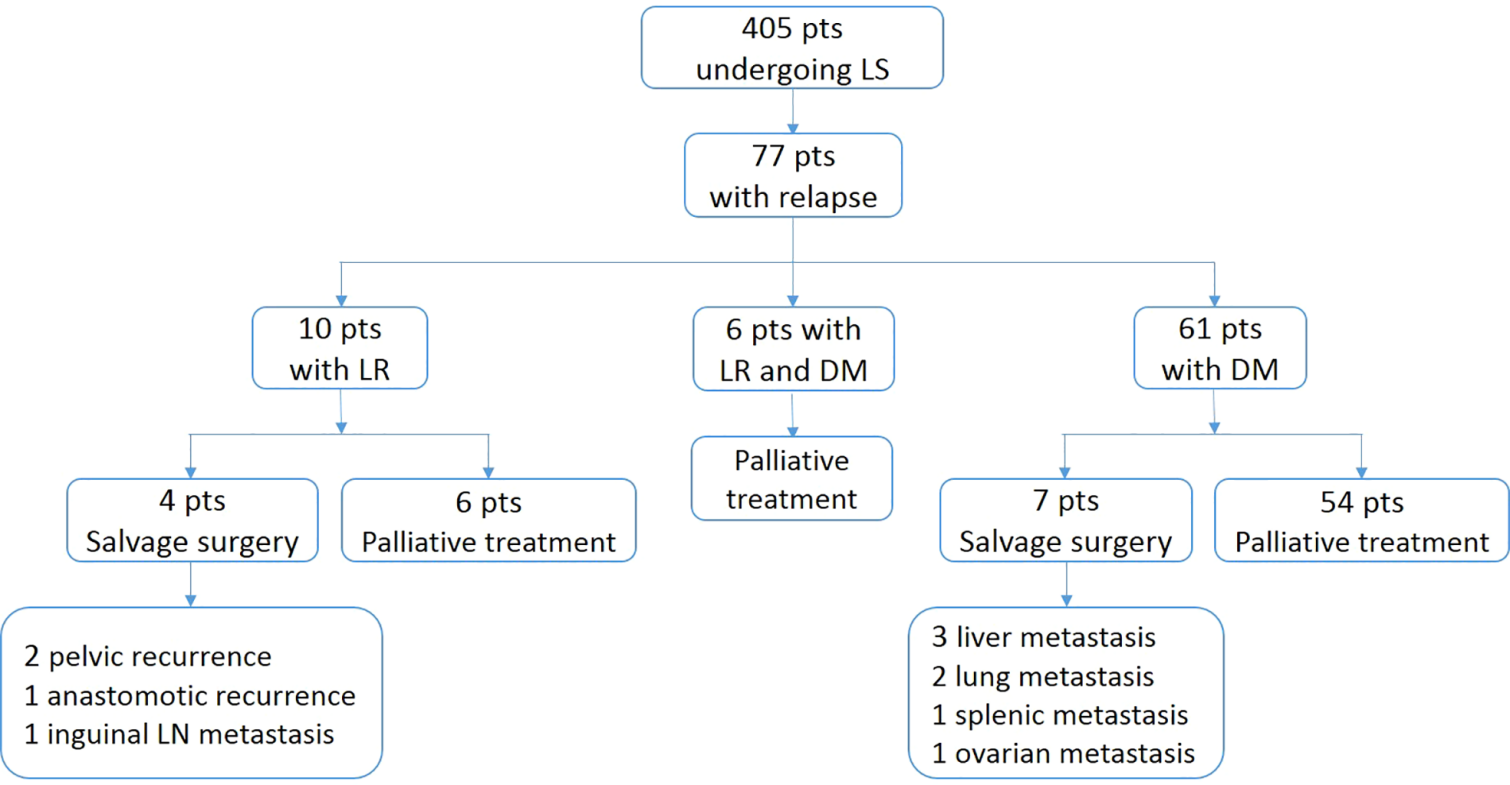
Flow diagram of patterns and treatment of patients with relapse. pts, patients; LS, laproscopic surgery; LR, locoregional recurrence; DM, distant metastasis.

The median follow-up time was 62 months (range, 1-125) for the entire cohort. Seventy-seven patients (19.0%) experienced disease recurrence (median time to relapse, 20 months): 10 (2.5%) had LR, 61 (15.1%) had DM, and 6 (1.5%) developed LR and DM synchronously (**Figure 1**). Of all patients with recurrence, 54.5% (n = 42) relapsed within the first 2 years, and 72.7% (n = 56) within the first 3 years. Three additional relapse, of whom one with LR and two with DM occurred after 5 years. Among the patients with DM alone, 27 cases had single-organ metastases, 34 developed multiple-organ metastases. The most common site of DM was the lung (n=39), followed by the liver (n=22), the distant lymph nodes (n=15), the bone (n=7) and the brain (n=6).

The results of the univariate analyses of risk factors for LR and DM are presented in **Table 3**. On multivariate analyses, only involved CRM (OR=13.708, 95% CI 3.478-54.026, P<0.001) was risk factor for LR, whereas elevated baseline level of CA19-9 (OR=3.299, 95% CI 1.461-7.449, P=0.032), advanced pN stage (OR=2.292, 95% CI 1.177-4.462, P=0.015) and harvested lymph nodes less than 12 (OR=2.418, 95% CI 1.245-4.695, P=0.009) were poor predictors for DM. (**Table 4**) The univariate analyses of risk factors for lung and liver metastasis are presented in Table supplementary. By multivariate analyses, results revealed that elevated baseline level of CEA (OR=2.540, 95% CI 1.224-5.271, P=0.012) and harvested lymph nodes less than 12 (OR=3.225, 95% CI 1.494-6.961, P=0.003) were independently associated with a higher risk of lung metastasis, while advanced pN stage (OR=3.152, 95% CI 1.256-7.910, P=0.014) significantly increased the risk of liver metastasis. (**Table 4**)

**Table 3:** Univariate analyses of risk factors for local and distant recurrence.

		Nunbers	LR		DM	
			n (%)	*P*	n (%)	*P*
Sex	Male	223	7(3.1)	0.357	38(17.0)	0.766
	Female	182	9(4.9)		29(15.9)	
Age (y)	≤60	209	8(3.8)	0.896	27(12.9)	0.044
	>60	196	8(4.1)		40(20.4)	
ASA	I	180	10(5.5)	0.164	23(12.6)	0.052
	II-III	225	6(2.7)		44(19.8)	
BMI (kg/m^2^)	<25	235	8(3.4)	0.509	38(16.2)	0.812
	≥25	170	8(4.7)		29(17.1)	
Location	Mid/low	266	12(4.5)	0.759	49(18.4)	0.161
	High	139	4(2.9)		18(12.9)	
Initial CEA (ng/ml)	≤5	282	9(3.2)	0.241	36(12.8)	0.002
	>5	123	7(5.7)		31(25.2)	
Initial CA19-9 (U/ml)	≤37	371	13(3.5)	0.142	52(14.0)	<0.001
	>37	34	3(8.8)		15(44.1)	
Type of surgery	Preserving	287	8(2.8)	0.069	43(15.0)	0.189
	Non-preserving	118	8(6.8)		24(20.3)	
Tumor differentiation	Well/moderate	339	11(3.2)	0.109	54(15.9)	0.452
	Poor	66	5(7.6)		13(19.7)	
pT stage	pT0-2	148	6(2.6)	0.935	18(12.2)	0.074
	pT3-4	257	10(5.9)		49(19.1)	
pN stage	pN0	235	5(2.1)	0.099	25(10.6)	<0.001
	pN1-2	170	9(5.3)		42(24.7)	
Lymphovascular invasion	Negative	351	11(3.1)	0.04	51(14.5)	0.007
	Positive	54	5(9.3)		16(29.6)	
Perineural invasion	Negative	380	15(3.9)	0.99	60(15.8)	0.118
	Positive	25	1(4.0)		7(28.0)	
Harvested lymph nodes	≥12	288	12(4.2)	0.727	41(14.2)	0.052
	<12	117	4(3.4)		26(22.2)	
DRM (cm)	≥1	385	16(4.2)	0.998	66(17.1)	0.186
	<1	20	0 (0)		1(5.0)	
CRM (mm)	>1	393	12(3.1)	<0.001	65(16.5)	0.991
	≤1	12	4(33.3)		2(16.7)	
Postoperative complications	Yes	51	2(3.9)	0.991	11(21.6)	0.304
	No	354	14(4.0)		56(15.8)	
Neoadjuvant therapy	Yes	115	4(3.5)	0.759	24(20.9)	0.142
	No	290	12(4.1)		43(14.8)	
Adjuvant chemotherapy	Yes	220	12(5.5)	0.102	41(18.6)	0.218
	No	185	4(2.2)		26(14.1)	

LR, locoregional recurrence; DM, distant metastasis; ASA, American Society of Anesthesiologists; BMI, body mass index; CEA, carcinoembryonic antigen; DRM, distal resection margin; CRM, circumferential resection margin.

**Table 4:** Multivariate analyses of risk factors for local and distant recurrence.

	OR	95% CI	*P*
LR (n=16)			
CRM (≤1 vs. >1mm)	13.708	3.478-54.026	<0.001
DM (n=67)			
Initial CA19-9 (>37 vs. ≤37 U/ml)	3.299	1.461-7.449	0.032
pN stage (N1-2 vs. N0)	2.292	1.177-4.462	0.015
Harvested lymph nodes (<12 vs. ≥12)	2.418	1.245-4.695	0.009
Lung metastasis (n=39)			
Initial CEA (>5 vs. ≤5 ng/ml)	2.632	1.284-5.393	0.008
Harvested lymph nodes (<12 vs. ≥12)	3.226	1.496-6.958	0.003
Liver metastasis (n=22)			
pN stage (N1-2 vs. N0)	3.152	1.256-7.910	0.014

List only variables statistically significant. LR, locoregional recurrence; DM, distant metastasis; CRM, circumferential resection margin; CEA, carcinoembryonic antigen; OR, odds ratio; CI, confidence interval.

### Salvage surgery on relapse cases

Patients with relapsed disease were included in the multidisciplinary therapy process. The treatment regimens of the recurrent patients are shown in **Figure 1**. Two patients with pelvic recurrence, one with anastomotic recurrence, and another with inguinal lymph node metastasis received radical excision, of whom only one was disease-free (median follow-up after initial relapse, 37 months). Three patents with liver metastasis, two with lung metastasis, one with splenic metastasis and another with ovarian metastasis were identified as resectable and underwent curative resection, of whom 5 patients were disease-free (median follow-up after initial relapse, 31 months). In all patients with recurrence, there was a significant difference in estimated 3-year survival between patients receiving salvage surgery and palliative treatment (90.9% vs. 20.5%; P=0.017).

### Disease-free survival and cancer-specific survival

The estimated 5-year DFS and CSS for the whole group was 80.2% and 83.1%, respectively. The univariate and multivariate analyses of prognostic factors for DFS and CSS are presented in **Tables 5**, **6**, respectively. Multivariate analyses revealed that elevated baseline level of CEA (HR=1.839, 95% CI 1.097-3.084, P=0.021) and CA19-9 (HR=2.169, 95% CI 1.164-4.043, P=0.015), advanced pN stage (HR=2.261, 95% CI 1.244-4.109, P=0.007) were independently associated with worse DFS. When this set of variables was used in the same model for CCS, it turned out that age older than 60 years (HR=1.839, 95% CI 1.113-3.041, P=0.017), non-preserving surgery (HR=1.866, 95% CI 1.127-3.087, P=0.015), poor differentiation (HR=1.998, 95% CI 1.151-3.467, P=0.014), advanced pN stage (HR=4.668, 95% CI 2.416-9.022, P<0.001) and positive lymphovascular invasion (HR=1.791, 95% CI 1.038-3.092, P=0.036) were independent adverse prognostic factors for CSS.

**Table 5:** Univariate and multivariate analyses of prognostic factors for disease-free survival (DFS).

		Numbers	Univariate		Multivariate		
			5-y DFS (%)	*P*	HR	95% CI	*P*
Sex	Male	223	81	0.66			
	Female	182	79.3				
Age(y)	≤60	209	84.3	0.052	1		0.145
	>60	196	75.9		1.456	0.878-2.414	
ASA	I	180	83.7	0.114			
	II-III	225	76.7				
BMI (kg/m2)	<25	235	80.5	0.968			
	≥25	170	79.9				
Location	Mid/low	266	77.7	0.096	1		0.687
	High	139	85.1		0.88	0.474-1.636	
Initial CEA (ng/ml)	≤5	282	85.3	0.001	1		0.021
	>5	123	68.7		1.839	1.097-3.084	
Initial CA19-9 (U/ml)	≤37	371	82.9	<0.001	1		0.015
	>37	34	51.2		2.169	1.164-4.043	
Type of surgery	Preserving	287	83.2	0.019	1		0.141
	Non-preserving	118	72.9		1.528	0.869-2.688	
Tumor differentiation	Well/moderate	339	81.6	0.108			
	Poor	66	72.2				
pT stage	pT0-2	148	85.8	0.077	1		0.759
	pT3-4	257	76.9		1.1	0.600-2.014	
pN stage	pN0	235	87.7	<0.001	1		0.007
	pN1-2	170	69.3		2.261	1.244-4.109	
Lymphovascular invasion	Negative	351	83	<0.001	1		0.065
	Positive	54	60.6		1.738	0.966-3.128	
Perineural invasion	Negative	380	81	0.085	1		0.359
	Positive	25	67.3		1.496	0.632-3.541	
Harvested lymph nodes	≥12	288	82.3	0.064	1		0.096
	<12	117	74.9		1.697	0.910-3.162	
DRM (cm)	≥1	385	79.5	0.11			
	<1	20	94.4				
CRM (mm)	>1	393	81.2	0.001	1		0.08
	≤1	12	50		2.191	0.909-5.280	
Postoperative complications	Yes	51	81.5	0.275			
	No	354	72.3				
Neoadjuvant therapy	Yes	115	74.4	0.049	1		0.238
	No	290	82.3		0.651	0.319-1.328	
Adjuvant chemotherapy	Yes	220	76.7	0.037	1		0.934
	No	185	84.5		0.977	0.564-1.693	

ASA, American Society of Anesthesiologists; BMI, body mass index; CEA, carcinoembryonic antigen; DRM, distal resection margin; CRM, circumferential resection margin; HR, hazard ratio; CI, confidence interval.

**Table 6:** Univariate and multivariate analyses of prognostic factors for cancer-specific survival (CSS).

		Numbers	Univariate		Multivariate		
			5-y CSS (%)	*P*	HR	95% CI	*P*
Sex	Male	223	83.6	0.787			
	Female	182	81.5				
Age(y)	≤60	209	87.1	0.074	1		0.017
	>60	196	79.1		1.839	1.113-3.041	
ASA	I	180	85.6	0.355			
	II-III	225	81.2				
BMI (kg/m2)	<25	235	81	0.112			
	≥25	170	86.1				
Location	Mid/low	266	81.4	0.158			
	High	139	86.6				
Initial CEA (ng/ml)	≤5	282	86.2	0.001	1		0.088
	>5	123	76.1		1.613	0.931-2.797	
Initial CA19-9 (U/ml)	≤37	371	85.8	<0.001	1		0.058
	>37	34	54.8		1.829	0.979-3.416	
Type of surgery	Preserving	287	85.9	0.09	1		0.015
	Non-preserving	118	76.2		1.866	1.127-3.087	
Tumor differentiation	Well/moderate	339	85.5	0.001	1		0.014
	Poor	66	70.2		1.998	1.151-3.467	
pT stage	pT0-2	148	90.9	0.006	1		0.822
	pT3-4	257	78.6		0.927	0.480-1.792	
pN stage	pN0	235	93.7	<0.001	1		<0.001
	pN1-2	170	68.3		4.668	2.416-9.022	
Lymphovascular invasion	Negative	351	87.2	<0.001	1		0.036
	Positive	54	57.1		1.791	1.038-3.092	
Perineural invasion	Negative	380	83.6	0.214			
	Positive	25	76.9				
Harvested lymph nodes	≥12	288	82.9	0.941			
	<12	117	84.2				
DRM (cm)	≥1	385	82.7	0.171			
	<1	20	92.9				
CRM (mm)	>1	393	83.2	0.393			
	≤1	12	80.2				
Postoperative complications	Yes	51	74.5	0.126			
	No	354	84.6				
Neoadjuvant therapy	Yes	115	82.6	0.529			
	No	290	83.6				
Adjuvant chemotherapy	Yes	220	82.8	0.554			
	No	185	83.5				

ASA, American Society of Anesthesiologists; BMI, body mass index; CEA, carcinoembryonic antigen; DRM, distal resection margin; CRM, circumferential resection margin; HR, hazard ratio; CI, confidence interval.

## Discussion

Rectal cancer has a high risk of relapse after surgery, so it is very important to explore the relevant factors affecting postoperative recurrence of rectal cancer. In the present study, we specifically focus on the long-term outcomes of laparoscopic rectal resection. The results showed that 19.0% of patients experienced disease recurrence with a median follow-up of 62 months, of which the majority occurred within 3 years. There were several clinicopathological factors may be relevant to disease recurrence after laparoscopic rectal resection.

In this study, LR was relatively less common after laparoscopic resection of rectal cancer, affecting approximately 4.0% of patients, which is consistent with previous studies mainly focused on open surgery (5, 7, 14). Involved CRM was identified as the only independent risk factor for LR. Several studies have shown a high frequency of CRM involvement in low rectal cancer (15–17), while others have indicated that involved CRM is one of the most important factors in determining surgical quality and predicting LR and long-term survival (18–20). Gosens et al. noted that LR accounted for only 8% among the patients with a clear CRM compared with 43% in case of positive CRM (18). Tilney et al. demonstrated that involved CRM was significantly relevant to increased LR and reduced CSS (20). In recent years, laparoscopic surgery for rectal cancer has been confirmed to have non-inferiority results compared with open surgery (9–11). The COLOR II study presented that both the laparoscopic and open surgery groups had an involved CRM rate of 10%, and the LR rate was 5% in both groups after 3 years (10). The COREAN study showed that CRM involvement accounted for 2.9% and 4.1% respectively in the laparoscopic surgery group and the open surgery group, and there was no significant difference in 3-year LR rate between the two groups (2.6% vs. 4.9%), even all patients included in this study had received NAT (9). Our results showed that the rate of CRM involvement after laparoscopic rectal resection was 3.0%, which is similar to previous studies from Asian countries (9, 21). LR occurred in only 3.1% of the patients with a clear CRM, but the LR rate in the patients with positive CRM was as high as 33.3%. In our opinion, laparoscopic surgery has a better surgical field of view than open surgery, especially when performed in the depth of the pelvic or applied to obese patients, which makes it easier for surgeons to identify anatomical landmarks and enter into the correct plane of anatomy. Therefore, laparoscopic surgery is theoretically capable of achieving better CRM outcomes, especially performed by well-skilled colorectal surgeons, but more research is needed to confirm the assumption.

Our study indicated that DM was more common than LR after surgery in rectal cancer, and lung metastasis was most frequently observed in patents with DM, this finding is also consistent with previous studies mainly focused on open surgery (5, 7, 22). The higher incidence of pulmonary recurrence in rectal cancer may be related to direct hematogenous diffusion into systemic circulation *via* the iliac veins. Moreover, NAT may also alter tumor biology in ways that limit liver metastasis or favor lung metastasis (5, 22). By multivariate analyses, elevated baseline level of CA19-9, advanced pN stage and harvested lymph nodes less than 12 were found to be independently associated with DM, and the first two were also independent prognostic factors for DFS, whereas advanced pN stage was associated with poor CSS. It is known that many tumor markers, such as CEA and CA19-9, are useful prognostic indicator to predict relapse and survival in rectal cancer patients (23–25). In addition to the association between baseline CA19-9 level and DM after laparoscopic resection of rectal cancer, our study showed that elevated baseline level of CEA was an independent risk factor for lung metastasis and predicted inferior DFS. In terms of pN stage, some studies have reported that it was an important factor to predict LR, DM and inferior survival (26, 27). In our study, advanced pN stage was not only related to higher risk of DM, but also the only independent risk factor for liver metastasis.

The current staging system of colorectal cancer presented by AJCC depends largely on the number of metastatic lymph nodes, which requires at least 12 lymph nodes harvested to guarantee accurate staging and avoid inadequate treatment. Even though TME principle and appropriate pathologic assessment were applied, many studies have indicated that NAT was usually relevant to decreased number of retrieved lymph nodes (28–30). However, other studies have demonstrated that suboptimal lymph node yield (<12) was independently associated with inferior overall survival irrespective of NAT (31, 32). In our cohort, the majority of patients (71.1%) had at least 12 lymph nodes dissected, and the median number of harvested lymph nodes for patients who received NAT and those who did not was 9 (range, 2-27) and 15.5 (range, 2-43), respectively. Multivariate analyses indicated that harvested lymph nodes less than 12 was an independent risk factor for DM and lung metastasis. Therefore, the dissection and detection of lymph nodes are very important for laparoscopic rectal resection. Not only should the surgeon ensure that the procedure meets the requirements of TME principle, but the pathologist should be more careful in the pathologic examination. Whereas other surgery-related factors, including sphincter-preserving or non-preserving surgery, distal resection margin, CRM status and postoperative morbidity, did not independently affect DM.

In recent years, the application of salvage surgery in patients with relapse after radical resection of colorectal cancer is increasing dramatically, and some studies have shown that patients receiving salvage surgery had significantly increased survival compared with nonresected patients (6, 8). In our study, curative-intent salvage surgery was implemented in 30% of patients (11 of 37) with single-site relapse. Of the 10 patients with isolated LR, 4 patients could be managed by salvage surgery, and only one was disease-free during follow-up. Among the 27 patients with single-organ DM, 7 patients received salvage surgery, and 5 patients were still disease-free. Patients receiving salvage surgery showed a significant survival advantage over those receiving palliative treatment, which was in accordance with previous published results (6, 8, 14). However, since the sample size of the study is small, it cannot provide enough strength for drawing any definitive conclusions. Ikoma et al. demonstrated that salvage surgery was associated with improved survival in patients with lung-only and liver-only recurrence, but not in those with LR (6). The authors speculated the reason might be that salvage surgery for LR was more difficult after NAT and high-quality TME, due to an increased risk of external central pelvic recurrence and more often need for extensive resection. Therefore, multidisciplinary consultation should be applied in the treatment of recurrent patients in order to single out candidates who can benefit from salvage surgery.

To our knowledge, this is one of very few studies discussing the patterns and predictors of recurrence after laparoscopic rectal resection. However, the present study has some limitations, since it is a retrospective study and the sample size is small, there must be inherent selection bias. Due to the small sample size and low incidence of specific events, such as involved CRM, the confidence interval of the variable was too wide, which reduced the statistical power to some extent. Second, because of the differences in patient compliance and economic conditions, treatment strategies for cases included in this study are not always appropriate. Some patients who should have received NAT before surgery underwent resection directly, which reduced the proportion of patients treated with NAT in this cohort. Finally, patients in this cohort were not followed up long enough, with a median follow-up of 62 months and individual patients less than 5 years, so the recurrence events may have been underestimated.

## Conclusion

In summary, our study demonstrated that distant metastasis was more common than locoregional recurrence after laparoscopic resection of rectal cancer, among which lung metastasis was the most frequent, and recurrence normally occurred within 3 years after surgery. Hence close surveillance was very important during this period. There were several clinicopathological factors related to tumor relapse after surgery. As for surgery-related factors, involved CRM was the only independent risk factor for LR, whereas harvested lymph nodes less than 12 was independently associated with DM and lung metastasis. These factors should be taken into account during operation. However, when laparoscopic surgery is performed by well-skilled colorectal surgeons, satisfactory CRM status and lymph nodes yield can be obtained. Based on the promising outcomes of salvage surgery, all patients with single-site recurrence should be carefully assessed by a multidisciplinary team and the possibility of salvage surgery should be considered.

## Data availability statement

The raw data supporting the conclusions of this article will be made available by the authors, without undue reservation.

## Ethics statement

The studies involving human participants were reviewed and approved by Research Ethics Committee of Peking University Cancer Hospital and Institute. The patients/participants provided their written informed consent to participate in this study. Written informed consent was obtained from the individual(s) for the publication of any potentially identifiable images or data included in this article.

## Author contributions

Study conception and design: HY and XS. Acquisition of data: HY, XW, CZ and ZY. Analysis and interpretation of data: HY, LC, JX, MC and BJ. Writing manuscript: HY and LC. All authors contributed to the article and approved the submitted version.

## Funding

This study was supported by National Natural Science Foundation of China (No. 82171720, 82173218, 81872022, 81672439). Our open access publication fees are available from the funding program mentioned above. The funders had no role in the study design, data collection, analysis and interpretation, publication decision or writing of the article.

## Conflict of interest

The authors declare that the research was conducted in the absence of any commercial or financial relationships that could be construed as a potential conflict of interest.

## Publisher’s note

All claims expressed in this article are solely those of the authors and do not necessarily represent those of their affiliated organizations, or those of the publisher, the editors and the reviewers. Any product that may be evaluated in this article, or claim that may be made by its manufacturer, is not guaranteed or endorsed by the publisher.
