# Supplementary material for: Patterns and predictors of recurrence after laparoscopic resection of rectal cancer
Source: Front Oncol. 2022 Oct 27;12:1034838. doi: 10.3389/fonc.2022.1034838 (PMC9647165; doi:10.3389/fonc.2022.1034838)
Supplement: Supplementary file 1 [file Table_1.doc]

**Table S Univariate analyses of risk factors for lung and liver** [**metastasis**](javascript:;)

|  |  | Nunbers | Lung [metastasis](javascript:;) | |  | Liver [metastasis](javascript:;) | |
| --- | --- | --- | --- | --- | --- | --- | --- |
|  |  |  | n (%) | *P* |  | n (%) | *P* |
| Sex    Age (y)    ASA    BMI (kg/m^2^)  Location    Initial CEA (ng/ml)  Initial CA19-9 (U/ml)  Type of surgery    Tumor differentiation    pT stage    pN stage    Lymphovascular invasion    Perineural invasion    Harvested lymph nodes    DRM (cm)  CRM (mm)  Postoperative complications  Neoadjuvant therapy    Adjuvant chemotherapy | Male  Female  ≤60  ＞60  I  II-III  ＜25  ≥25  Mid/low  High  ≤5  ＞5  ≤37  ＞37  Preserving Non-preserving  Well/moderate  Poor  pT0-2  pT3-4  pN0  pN1-2  Negative  Positive  Negative  Positive  ≥12  ＜12  ≥1  ＜1  ＞1  ≤1  Yes  No  Yes  No  Yes  No | 223  182  209  196  180  225  235  170  266  139  282  123  371  34  287  118  339  66  148  257  235  170  351  54  380  25  288  117  385  20  393  12  51  354  115  290  220  185 | 23(10.3)  16(8.8)  16(7.7)  23(11.7)  13(7.2)  26(11.6)  21(8.9)  18(10.6)  30(11.3)  9(6.5)  19(6.7)  20(16.3)  33(8.9)  6(17.6)  25(8.7)  14(11.9)  32(9.4)  7(10.6)  8(5.4)  31(12.1)  16(6.8)  23(13.5)  32(9.1)  7(13.0)  35(9.2)  4(16.0)  23(8.0)  16(13.7)  38(9.9)  1(5.0)  38(9.7)  1(8.3)  6(11.8)  33(9.3)  15(13.0)  24(8.3)  23(10.5)  16(8.6) | 0.606  0.167  0.145  0.578  0.124  0.004  0.105  0.330  0.769  0.033  0.026  0.375  0.272  0.082  0.481  0.877  0.581  0.146  0.540 |  | 15(6.7)  7(3.8)  9(4.3)  13(6.6)  10(5.6)  12(5.3)  14(6.0)  8(4.7)  16(6.0)  6(4.3)  11(3.9)  11(8.9)  17(4.6)  5(14.7)  17(5.9)  5(4.2)  21(6.2)  1(1.5)  5(3.4)  17(6.6)  7(3.0)  15(8.8)  16(4.6)  6(11.1)  19(5.0)  3(12.0)  14(4.9)  8(6.8)  22(5.7)  0(0)  22(5.6)  0(0)  4(7.8)  18(5.1)  6(5.2)  16(5.5)  16(7.3)  6(3.2) | 0.209  0.305  0.922  0.584  0.476  0.045  0.019  0.498  0.158  0.174  0.014  0.056  0.149  0.429  0.998  0.999  0.420  0.904  0.082 |

LR, locoregional recurrence; DM, d[istant](javascript:;) [metastasis](javascript:;); ASA, American Society of Anesthesiologists; BMI, body mass index; CEA, carcinoembryonic antigen; DRM, distal resection margin; CRM, circumferential resection margin
